# Preparation of Sulfonated Poly(arylene ether)/SiO_2_ Composite Membranes with Enhanced Proton Selectivity for Vanadium Redox Flow Batteries

**DOI:** 10.3390/molecules28073130

**Published:** 2023-03-31

**Authors:** Zhoulin Ye, Nanjie Chen, Zigui Zheng, Lei Xiong, Dongyang Chen

**Affiliations:** School of Materials Science and Engineering, Fuzhou University, Fuzhou 350116, China

**Keywords:** proton exchange membrane, sulfonated poly(arylene ether), composite, proton conductivity, vanadium permeability

## Abstract

Proton exchange membranes (PEMs) are an important type of vanadium redox flow battery (VRFB) separator that play the key role of separating positive and negative electrolytes while transporting protons. In order to lower the vanadium ion permeability and improve the proton selectivity of PEMs for enhancing the Coulombic efficiency of VRFBs, herein, various amounts of nano-sized SiO_2_ particles were introduced into a previously optimized sulfonated poly(arylene ether) (SPAE) PEMs through the acid-catalyzed sol-gel reaction of tetraethyl orthosilicate (TEOS). The successful incorporation of SiO_2_ was confirmed by FT-IR spectra. The scanning electron microscopy (SEM) images revealed that the SiO_2_ particles were well distributed in the SPAE membrane. The ion exchange capacity, water uptake, and swelling ratio of the PEMs were decreased with the increasing amount of SiO_2_, while the mechanical properties and thermal stability were improved significantly. The proton conductivity was reduced gradually from 93.4 to 76.9 mS cm^−1^ at room temperature as the loading amount of SiO_2_ was increased from 0 to 16 wt.%; however, the VO^2+^ permeability was decreased dramatically after the incorporation of SiO_2_ and reached a minimum value of 2.57 × 10^−12^ m^2^ s^−1^ at 12 wt.% of SiO_2._ As a result, the H^+^/VO^2+^ selectivity achieved a maximum value of 51.82 S min cm^−3^ for the composite PEM containing 12 wt.% of SiO_2_. This study demonstrates that the properties of PEMs can be largely tuned by the introduction of SiO_2_ with low cost for VRFB applications.

## 1. Introduction

Vanadium redox flow batteries (VRFBs), owing to their outstanding merits such as low cost, fast response, high efficiency, good cycling stability, and promising application in large-scale energy storage, have attracted extensive attention over the past decades as the most promising large-scale energy storage units [1,2,3]. In VRFBs, the proton exchange membrane (PEM) is an important separator between the catholyte and anolyte that prevents the cross-mixing of electrolytes while allowing the migration of conductive ions (H^+^ or SO_4_^2−^) to complete the electrical circuit. An ideal PEM should possess high ion conductivity, low vanadium permeability, robust mechanical strength, and good chemical stability [4,5]. Nafion membranes, a typical perfluorinated sulfonic acid membrane developed by DuPont, have been widely acknowledged as the benchmark material for VRFBs due to their high proton conductivity and excellent chemical stability. However, the challenge of achieving high vanadium permeability in Nafion membranes, as well as their low ion selectivity and high cost, still restrict their large-scale application in VRFBs [6,7,8,9]. Alternatively, sulfonated fluorinated aromatic polymers, such as sulfonated poly(arylene ether ketone) (SPAEK) [10,11,12], sulfonated polyimide (SPI) [13,14,15], and sulfonated polysulfone (SPSF) [16,17], have attracted much attention due to their excellent comprehensive performance, low cost, and easy preparation [18,19,20,21,22].

Organic–inorganic composite PEMs combine the advantages of organic polymers and inorganic fillers [23]. They are usually prepared by incorporating inorganic filling materials such as SiO_2_, TiO_2_, and graphene oxide (GO) into PEMs. Many research groups have developed this type of composite PEM with excellent performance. For example, Xi et al. [24] prepared Nafion/SiO_2_ composite membrane using the sol–gel method, and the doped SiO_2_ particles (9.2 wt.%) were distributed in the hydrophilic channel network of Nafion 117. Compared with those of unmodified Nafion 117, slightly reduced water uptake (26.0% vs. 21.5%) and proton conductivity (58.7 mS cm^−1^ vs. 56.2 mS cm^−1^) were achieved for the composite PEMs with significantly reduced vanadium ion permeability under similar IEC. The VRFB assembled with Nafion/SiO_2_ hybrid membranes showed higher Coulombic efficiency and energy efficiency together with a lower self-discharge rate than the VRFB assembled with Nafion 117. Hossain and Aziz et al. [25] prepared SPAEK/Ce_2_Zr_2_O_7_ composite membrane by doping Ce_2_Zr_2_O_7_ nano-oxide into SPAEK solution. The vanadium ion permeability of the composite membrane was 27 and 12 times lower than those of the pristine Nafion 212 and SPAEK membranes, respectively. These literature studies show that the physicochemical properties of PEMs can be improved by introducing inorganic nanofillers. The inorganic nanoparticles not only have stable chemical properties but also can be easily mixed into the ion transporting channel of PEMs due to their hydrophilicity and small size, which can effectively inhibit the permeation of vanadium ions and improve the proton selectivity of PEMs [26,27,28,29,30,31,32,33,34].

For the real commercialization of VRFBs, the cost of its separator is critically important. While many high-performance PEMs can be prepared from specially designed polymers and fillers [35,36,37,38,39], the cost associated with the full synthesis process may be very high. In addition, those syntheses may not be easily scaled up. To address these challenges, herein, we adopt an economic way to incorporate SiO_2_ into a previously optimized sulfonated poly(arylene ether) (SPAE) PEM [40] via the acid-catalyzed sol-gel reaction of TEOS. SPAE was selected because of its higher Coulombic efficiency and energy efficiency compared to Nafion 212 when assembled in VRFBs, which is realized by its ion-clustered macromolecular structure with distinct phase separation. SiO_2_ was selected as a dopant due to its ease of synthesis, low cost, and excellent chemical stability in the electrolytes of VRFBs. The influence of the amount of SiO_2_ on the ion exchange capacity, water uptake, swelling ratio, tensile strength, elongation-at-break, proton conductivity, vanadium ion permeability, and thermal stability of the membranes was investigated in detail.

## 2. Results and Discussion

### 2.1. Preparation of the SPAE/SiO_2_ Composite PEMs

**Figure 1:**
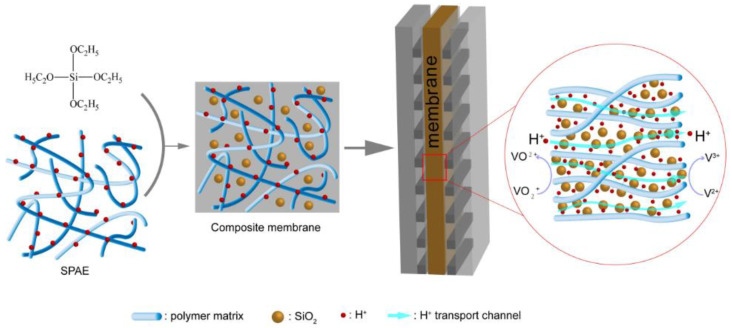
Schematic of VRFB based on the prepared SPAE/SiO_2_ composite PEMs.

Due to the high surface energy, nano-SiO_2_ agglomerates easily, making it difficult to disperse uniformly in a polymer matrix. Herein, various amounts of SiO_2_ were introduced into SPAE by acid-catalyzed sol-gel reaction of TEOS, as depicted in Figure 1. After ultrasonic dispersion and mechanical agitation, uniform mixtures were formed, and the SPAE/SiO_2_ composite PEMs were successfully cast from these mixtures. The hydrophilic SiO_2_ particles were expected to fill in the hydrophilic channels of SPAE, thus increasing the tortuosity of the hydrophilic channels and depressing the permeability of vanadium ions to mitigate the capacity decay and increase the Coulombic efficiency of VRFBs.

**Figure 2:**
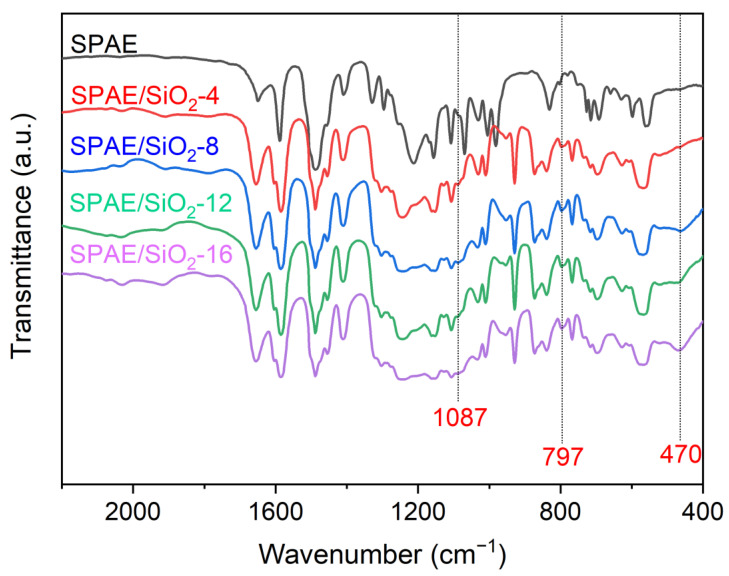
FT-IR spectra of the SPAE and SPAE/SiO_2_ composite PEMs.

The incorporation of SiO_2_ into SPAE was confirmed by FT-IR spectra, as shown in Figure 2. The characteristic uptake peaks of SiO_2_ appeared at 1087 cm^−1^ (asymmetric stretching of Si-O-Si), 797 and 470 cm^−1^ (symmetric stretching of Si-O), confirming the successful incorporation of SiO_2_ into SPAE. It should be noted that the absorption peaks of SPAE are almost unchanged after the incorporation of SiO_2_, even though the -OH groups on SiO_2_ may form hydrogen bonds with the -SO_3_H on SPAE. This may be because the hydrogen bonding within SPAE is much stronger than the hydrogen bonding between SiO_2_ and SPAE.

### 2.2. Microstructure of the SPAE/SiO_2_ Composite PEMs

**Figure 3:**
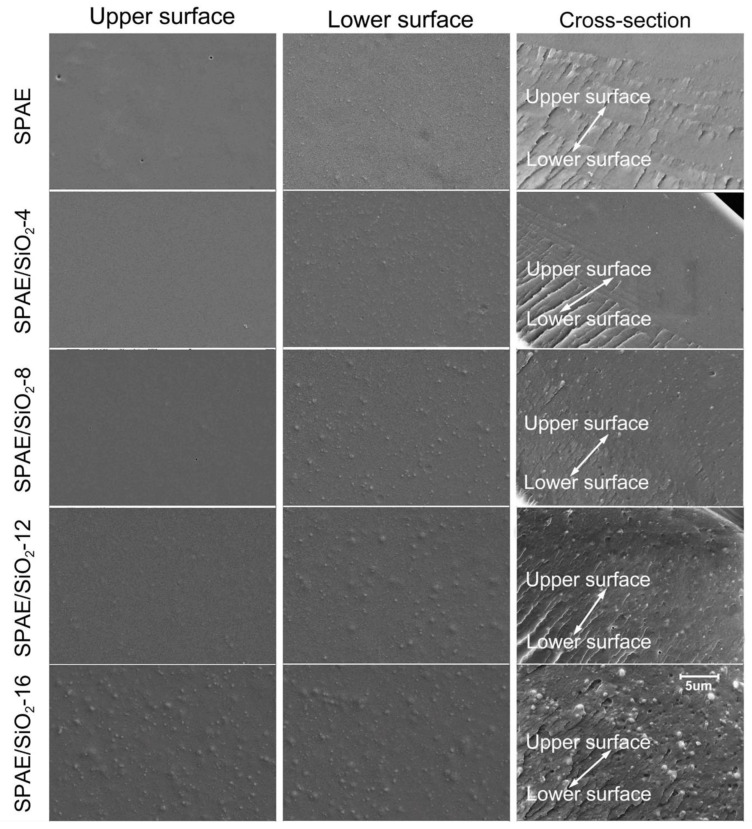
SEM images of the SPAE and SPAE/SiO_2_ composite PEMs.

The upper surface, lower surface, and cross-section morphology of the pristine SPAE and the SPAE/SiO_2_ composite PEMs were investigated by SEM, as shown in Figure 3. The surface in contact with air during casting was the upper surface, and the surface in contact with the glass substrate was the lower surface. The SiO_2_ particles can be seen in the SEM images of the SPAE/SiO_2_ composite PEMs without obvious agglomeration, even though the size of SiO_2_ particles increases with the increase of SiO_2_ content. Slight sedimentation of SiO_2_ particles on the lower surface can be seen, which can be ascribed to gravity during the casting process. The introduction of SiO_2_ particles into the polymer matrix does not introduce porosity, indicating good contact between the polymer matrix and filler. These results suggest that the SiO_2_ particles are well dispersed in the SPAE/SiO_2_ composite PEMs.

### 2.3. IEC, Water Uptake, and Swelling Ratio

The IEC, water uptake, and swelling ratio of the prepared membranes at room temperature are listed in Table 1. The IEC of the pristine SPAE is 1.97 mmol g^−1^, and that of the SPAE/SiO_2_ composite PEMs varies in the range of 1.70–1.90 mmol g^−1^ due to the addition of different mass fractions of SiO_2_. The water uptake and swelling ratio of the SPAE/SiO_2_ composite PEMs decreased gradually with the increase of SiO_2_ content, which was because of the lowered IEC after the incorporation of SiO_2_. Specifically, the water uptake and swelling ratio of the SPAE were 25.6 and 13.3%, respectively, and decreased to 15.2 and 8.7%, respectively, for the SPAE/SiO_2_-16 (IEC = 1.70 mmol g^−1^).

**Figure 4:**
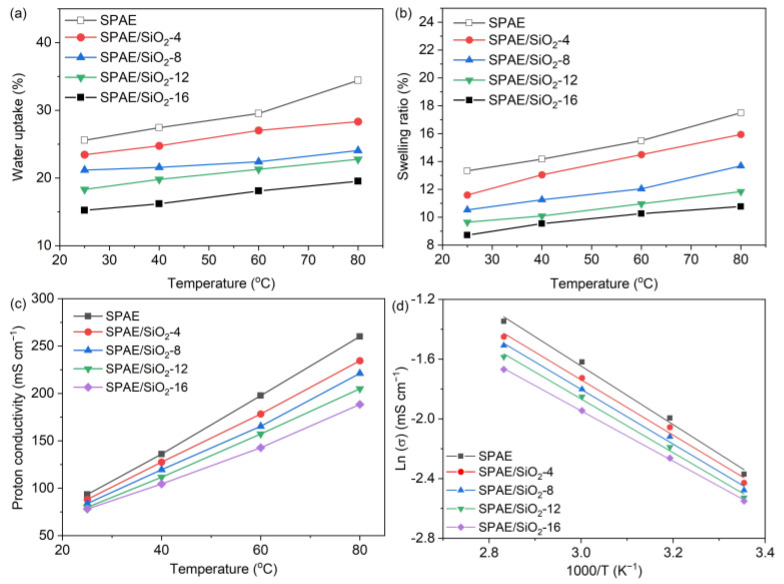
(**a**) Water uptake and (**b**) swelling ratio of the SPAE and SPAE/SiO_2_ composite PEMs as a function of temperature, (**c**) proton conductivity, and (**d**) Arrhenius plots of the SPAE and SPAE/SiO_2_ composite PEMs.

The water uptake and swelling ratio of pristine SPAE and SPAE/SiO_2_ composite PEMs as a function of temperature are plotted in Figure 4. It can be seen that all of the prepared membranes absorb more water at elevated temperatures, leading to higher water uptake (Figure 4a) and swelling ratio (Figure 4b). This is a common physicochemical phenomenon for ionomers as the interaction between water and ionomers is enhanced at high temperatures [41]. If the mass of SiO_2_ from the composite membrane is deducted to calculate the actual water uptake of the polymer itself, the actual water uptake of the polymer component in the SPAE/SiO_2_-16 is 17.6%, which is also much lower than the pristine SPAE (25.6%). The result implies that doping SiO_2_ can significantly inhibit the water uptake of the membrane. Since there is no chemical bonding between SPAE and SiO_2_, the reduced water uptake may be ascribed to the surface confinement of SiO_2_, which restricts the polymer chains from being swelled by water molecules.

### 2.4. Proton Conductivity and Area Resistance

Proton conductivity is a core property of PEMs, which is mainly affected by IEC, ion distribution, and water uptake [42]. The proton conductivity (σ) of the prepared membranes was evaluated in deionized water at various temperatures; the relationship between proton conductivity and temperature is shown in Figure 4c,d. In general, the proton conductivity increases with the increase in temperature owing to the increased free volume of the matrix and the activated proton migration at higher temperatures. The proton conductivity of the SPAE/SiO_2_ composite PEMs decreases with the increase of SiO_2_ content. At room temperature, the proton conductivity decreases from 93.4 mS cm^−1^ for SPAE to 76.9 mS cm^−1^ for SPAE/SiO_2_-16, as listed in Table 2. For comparison, the proton conductivity of Nafion 212 is 85.0 mS cm^−1^ at room temperature, which is slightly lower than that of SPAE. The apparent activation energy (E_a_) was calculated from the slope of Arrhenius plots (ln σ vs. 1000/T curves), as shown in Figure 4d [43], and the results are listed in Table 2. The E_a_ values of the composite PEMs are in the range of 14.03–15.58 kJ mol^−1^, indicating that the incorporation of SiO_2_ into the membrane does not increase the E_a_; instead, it lowers the E_a_ slightly. This implies that the hydrophilic SiO_2_ may facilitate the dissociation and hopping of protons. The area resistance of the prepared membranes increases with the increase of SiO_2_ content, as listed in Table 2. With the increase of SiO_2_ content from 4 wt.% to 16 wt.%, the area resistance of the SPAE/SiO_2_ composite PEMs increases from 0.87 Ω cm^2^ to 1.59 Ω cm^2^, suggesting that the introduction of SiO_2_ sacrifices the area resistance.

### 2.5. Thermal and Mechanical Properties

**Figure 5:**
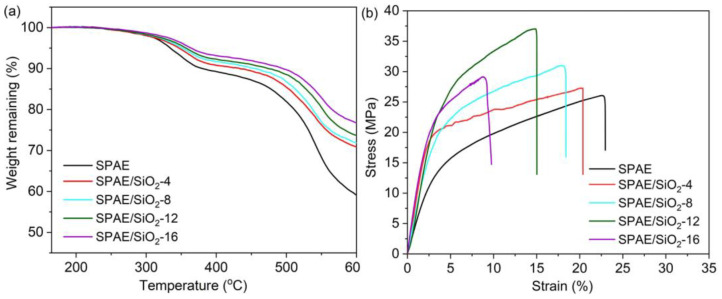
(**a**) TGA curves and (**b**) strain–stress curves of the SPAE and SPAE/SiO_2_ composite PEMs.

The thermal stability of the prepared membranes was investigated by thermogravimetric analysis (TGA) under N_2_ atmosphere. Prior to measurements, all samples were held at 150 °C for 30 min. to remove any absorbed water and residual solvents. As shown in Figure 5a, two steps of weight loss can be found for the pristine SPAE and SPAE/SiO_2_ composite PEMs. The first step of weight loss (300~360 °C) can be attributed to the decomposition of sulfonic acid groups, while the second step of weight loss (>480 °C) can be attributed to the decomposition of the polymer backbone. The 5% weight-loss temperature (T_d-5%_) for SPAE is 345 °C, and the T_d-5%_ of the SPAE/SiO_2_ composite PEMs increases with the increase of SiO_2_ content. Therefore, the introduction of the thermally stable SiO_2_ enhances the thermal stability of the PEM.

The mechanical strength of PEMs is critically important for practical applications. The stress-strain curves of the SPAE and SPAE/SiO_2_ composite PEMs are shown in Figure 5b, and the corresponding tensile strength and elongation at break are listed in Table 1. The tensile strength of the SPAE/SiO_2_ composite PEMs increases as the SiO_2_ content is increased from 0 to 12 wt.%, then decreases as the SiO_2_ content is further increased to 16 wt.%. The sudden decrease in tensile strength of SPAE/SiO_2_-16 may be ascribed to the relative aggregation of nano-sized SiO_2_ at this doping amount. The elongation at break of the SPAE/SiO_2_ composite PEMs decreases with the increase of SiO_2_ content. Therefore, the introduction of SiO_2_ generally increases the stiffness of the membrane. Since the SPAE matrix is very flexible, the introduction of a moderate amount of SiO_2_ is still advantageous for VRFB applications.

### 2.6. VO^2+^ Permeability and Ion Selectivity

**Figure 6:**
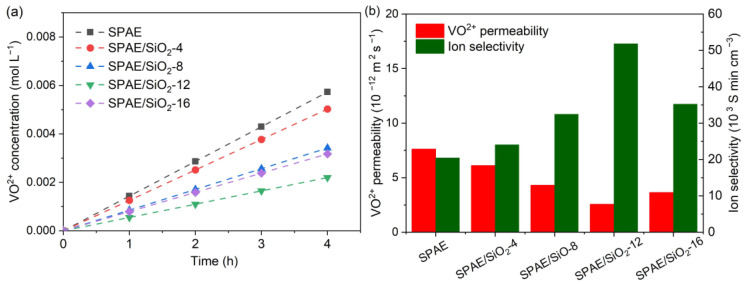
(**a**) Permeated VO^2+^ concentration as a function of time and (**b**) VO^2+^ permeability and ion selectivity of the SPAE and SPAE/SiO_2_ composite PEMs.

The vanadium ion permeability of PEMs affects the Coulombic efficiency and capacity decay of the VRFBs [44]. Since both vanadium ions and protons are cations, the vanadium ion permeability of PEMs is usually significant. Doped nanohybrids can fill in the ion transport channels of PEMs and act as barriers to vanadium ions by increasing the tortuosity of the channels; therefore, they have received much attention [45]. Since the Stokes radius of hydrated protons is significantly smaller than the hydrated vanadium ions, the proton conductivity can be much less influenced by the introduction of nanofillers [46]. The permeation of VO^2+^ through the SPAE and SPAE/SiO_2_ composite PEMs was recorded, as shown in Figure 6a. Linear permeation of VO^2+^ as a function of time can be found, which follows Fick’s law of diffusion. The VO^2+^ permeabilities of the SPAE and SPAE/SiO_2_ composite PEMs were calculated, as shown in Table 2 and Figure 6b. The VO^2+^ permeability of SPAE is 7.62 × 10^−12^ m^2^ s^−1^, which is greater than that of Nafion 212 (5.36 × 10^−12^ m^2^ s^−1^). The VO^2+^ permeability of the composite PEMs decreases gradually with the introduction of SiO_2_ until the SiO_2_ content is 12% and then increases slightly when the SiO_2_ content is further increased to 16%. SPAE/SiO_2_-12 has the lowest VO^2+^ permeability of 2.57 × 10^−12^ m^2^ s^−1^. For SPAE/SiO_2_-16, the VO^2+^ permeability is 3.64 × 10^−12^ m^2^ s^−1^, which may be ascribed to the relative aggregation of SiO_2_ in the membrane, resulting in a lowered blocking capability. Therefore, the introduction of SiO_2_ is effective in suppressing the permeation of VO^2+^, and the best doping content is 12% for the SPAE/SiO_2_ composite PEMs. Since the permeations of V^2+^, V^3+^, and VO_2_^+^ are similar to the permeation of VO^2+^, the VO^2+^ permeability is usually selected as a representative parameter for PEM evaluations [10]. For practical applications, the permeation of all four valence states of vanadium ions, driven not only by the concentration gradient but also by the electric field, should be considered together.

The ion (H^+^/VO^2+^) selectivities of the SPAE and SPAE/SiO_2_ composite PEMs are shown in Table 2 and Figure 6b. While the SPAE/SiO_2_ composite PEMs have slightly lower proton conductivity than the pristine SPAE, their VO^2+^ permeability is remarkably lower. Therefore, their ion selectivity is much higher than the pristine SPAE. This can be attributed to the increased tortuosity of the proton conducting channels for the composite PEMs. The ion selectivity of the SPAE/SiO_2_ composite PEMs first increases with the increase of SiO_2_ content, from 24.06 S min cm^−3^ for SPAE/SiO_2_-4 to 51.82 S min cm^−3^ for SPAE/SiO_2_-12 at room temperature, and then decreases to 35.21 S min cm^−3^. The SPAE/SiO_2_-12 has the highest ion selectivity (nearly 1.5 times higher than the pristine SPAE) because of its balanced proton conductivity and VO^2+^ permeability. For comparison, the ion selectivity of Nafion 212 is only 15.84 S min cm^−3^. It has been reported that the incorporation of SiO_2_ [47], amino-SiO_2_ [48], sulfated ZrO_2_ [49], sulfonated graphene oxide [50], and phosphotungstic acid immobilized Kevlar fibers [51] can increase the ion selectivity of PEMs for VRFB applications. The ion selectivity of SPAE/SiO_2_-12 is comparable to or even higher than those of the composite PEMs in the cited literature. Given the ease of preparation and low cost, SPAE/SiO_2_-12 is an attractive candidate PEM for the commercialization and long-term operation of VRFBs.

### 2.7. Oxidative Stability

**Figure 7:**
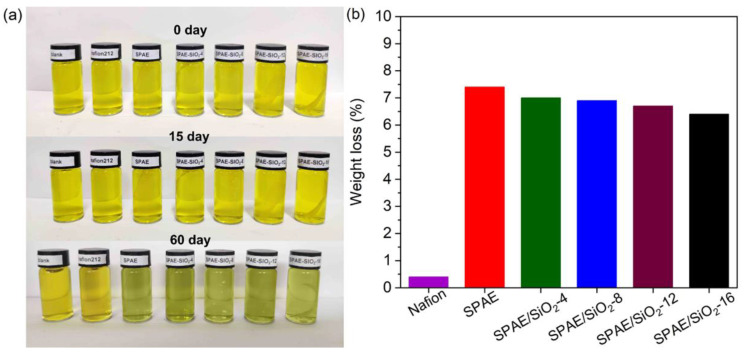
(**a**) Digital photos of Nafion 212, SPAE, and SPAE/SiO_2_ composite PEMs in 1.0 M VO^2+^ + 2.0 M H_2_SO_4_ solution at the 0, 15, and 60 days; (**b**) weight loss of Nafion 212, SPAE, and SPAE/SiO_2_ composite PEMs after 30 days of immersion.

The oxidative stability of the PEMs can be evaluated ex situ by immersing the samples in the oxidative VRFB catholyte [52]. The color change of the catholyte and the weight change of the samples are two important indications of relative stability. As shown in Figure 7a, the initial color of the VRFB catholyte is yellow. After 15 days of immersions with Nafion 212, SPAE, and SPAE/SiO_2_ composite PEMs, the colors of the VRFB catholytes are nearly unchanged. When the immersion time is extended to 60 days, the color of the VRFB catholyte with Nafion 212 is still yellow; however, the colors of the VRFB catholytes with SPAE and SPAE/SiO_2_ composite PEMs change to light blue, suggesting that some degradation has taken place. The colors are almost the same for the SPAE and SPAE/SiO_2_ composite PEMs, suggesting that their degradation rate is similar. After 60 days of immersion, all the samples remain intact, and the weight loss of the SPAE is 7.4%, while that of the composite PEMs range from 6.4 to 7.0%. The smaller weight loss of the composite PEMs may be attributed to their reduced water uptake, which lowers the amount of the oxidative VO_2_^+^ species diffusing into the membranes to carry out the degradation inside.

## 3. Experimental Section

### 3.1. Materials

Sulfonated poly(arylene ether) (SPAE) with an ion exchange capacity (IEC) of 1.97 mmol g^−1^ was synthesized according to our previous report [40]. Other chemicals were obtained from commercial sources and used as received.

### 3.2. Preparation of SPAE/SiO_2_ Composite PEMs

SiO_2_ was introduced into SPAE solution by sol-gel reaction of TEOS [53], and the doping degree was controlled by adjusting the added amount of TEOS. The specific steps are as follows. Firstly, SPAE was acidified with 1 M sulfuric acid solution and purified by dissolving in *N,N′*-dimethylacetamide (DMAc), filtering to remove insoluble impurities, and drying in an oven. Then, 1.0 g of SPAE was dissolved in 20 mL of DMAc, and a certain amount of TEOS was added dropwise with a liquid transfer gun and ultrasonicated for 0.5 h. The pH of the mixture was adjusted to 4 by adding 0.1 M sulfuric acid solution. The mixture was stirred for 24 h, cast on a horizontal glass plate, dried at 80 °C for 12 h, then dried at 80 °C under vacuum for another 12 h. Finally, the glass plate was immersed in deionized water, and the membrane was peeled off. The membranes were noted as SPAE/SiO_2_−x composite PEMs, where x represents the mass percentage of SiO_2_. These composite PEMs were acidified with 1 M sulfuric acid solution and stored in deionized water for further use. All the composite PEMs with different doping levels were prepared with the same polymer mass and the same size of the glass substrate, so the thickness of the membranes showed little difference, all lying in the range of 50–55 μm.

### 3.3. Characterizations

Fourier transform infrared spectroscopy (FT-IR, Nicolet 5700, Thermo Fisher, MA, USA) was recorded at 4000–400 cm^−1^, 32 times s^−1^, resolution 0.2 cm^−1^. Thermal stabilities were analyzed using a thermogravimetric analyzer (TGA, SDT-Q600, TA Instruments, New Castle, DE, USA) with a heating rate of 10 °C min^−1^ under a nitrogen atmosphere (flow rate: 20 mL min^−1^), and the samples were conditioned at 150 °C for 30 min. to remove the residual solvent and water. The morphologies of the membranes were investigated by scanning electron microscopy (SEM, SUPRA 55, Zeiss, Jena, Germany). The samples were sprayed with gold to increase conductivity.

#### 3.3.1. Water Uptake and Swelling Ratio

Water uptake and swelling ratio of membranes were measured at 25, 40, 60, and 80 °C. The samples were immersed in deionized water and equilibrated at the corresponding testing temperature for 24 h. The water uptake and swelling ratio were then calculated from the following equations:

(1) $$Water\;uptake\left(\%\right)=\frac{W_{wet}-W_{dry}}{W_{dry}}\times 100$$

(2) $$Swelling\;ratio\left(\%\right)=\frac{L_{wet}-L_{dry}}{L_{dry}}\times 100$$

where $W_{wet}$ and $W_{dry}$ are the wet and dry mass of the samples, respectively; $L_{wet}$ and $L_{dry}$ are the wet and dry lengths of the samples, respectively.

#### 3.3.2. Oxidative Stability

Oxidative stability was evaluated under acidic conditions by immersing the membranes in 1.0 M VO^2+^ + 2.0 M H_2_SO_4_ solution at room temperature for 60 days and then recording the weight loss of the sample and the color change of the solution [54].

#### 3.3.3. Mechanical Properties

Mechanical properties were measured by a universal stretching machine (UTM 6502X, SUNS universal testing instrument, Shenzhen, China). Rectangular samples were taken out from deionized water and measured immediately with a stretching rate of 5 mm min^−1^.

#### 3.3.4. Ion Exchange Capacity (IEC)

The IEC values of composite membranes were measured by acid-base titration. The composite membrane was immersed in 1 M of Na_2_SO_4_ solution for 24 h, followed by titration with 0.1 M NaOH solution using phenolphthalein as the indicator. The volume of consumed NaOH solution was recorded. The formula was as follows.

(3) $$IEC=\frac{\Delta V_{NaOH}\times C_{NaOH}}{W_{dry}}$$

where $\Delta V_{NaOH}$ is the consumed volume of NaOH solution, $C_{NaOH}$ is the concentration of NaOH solution, and $W_{dry}$ is the weight of the dry sample.

#### 3.3.5. Proton Conductivity

The proton conductivities were measured by AC impedance spectroscopy using an impedance/gain phase analyzer (Solartron 1260, Ametek, Newark, DE, USA) in a frequency range from 1 to 10 MHz [55,56]. The conductivities were calculated as follows [57,58].

(4) $$\sigma =\frac{L}{RS}$$

where L is the distance between two electrodes, R is the resistance of hybrid membranes, and S is the transverse area of the composite membranes.

#### 3.3.6. Area Resistance

The area resistances were measured according to the method reported in the literature [59]. Before testing, the composite membranes were soaked in 1.0 M VO^2+^ + 2.0 M H_2_SO_4_ solution for 24 h. When starting to test, the membrane was clamped between two compartments filled with the 1.0 M VO^2+^ + 2.0 M H_2_SO_4_ solution. The effective measuring area of the membrane (S) was 2.41 cm^2^. When testing the resistance of the device with membrane ($R_{1}$) and without membrane ($R_{2}$), respectively, the area resistances (AR) were calculated as follows.

(5) $$AR=\left(R_{1}-R_{2}\right)\times S$$

#### 3.3.7. VO^2+^ Permeability and Ion Selectivity

The VO^2+^ permeabilities were measured according to the method reported in the literature [60]. The membrane was clamped between a two-compartment diffusion cell (containing 80 mL of 1.0 M VOSO_4_ + 2.0 M H_2_SO_4_ solution and 80 mL of 1.0 M MgSO_4_ + 2.0 M H_2_SO_4_ solution). A volume of 1 mL of solution from the MgSO_4_ cell was taken out every 1 h at room temperature and titrated using an ultraviolet spectrophotometer (UV−5800 HPC, METASH, Shanghai, China, wavelength: 765 nm) to give the concentration of VO^2+^. The VO^2+^ permeabilities were calculated using the Formula (6) [61,62].

(6) $$V_{B}\frac{dC_{B}\left(t\right)}{dt}=A\frac{D}{L}\left(C_{A}-C_{B}\left(t\right)\right)$$

where A (cm^2^) and L (cm) are effective membrane area and the membrane thickness, t (s) is testing time, D is the VO^2+^ permeabilities, $C_{A}$ and $C_{B}$ (mol/L) are initial concentration of VO^2+^ in VOSO_4_ and MgSO_4_ solution, respectively, and $V_{B}$ (mL) is the volume of MgSO_4_. The ion selectivity was defined as the ratio of proton conductivity over VO^2+^ permeability.

## 4. Conclusions

Various amounts of nano-sized SiO_2_ particles were successfully introduced into a previously optimized sulfonated poly(arylene ether) (SPAE) PEM through the acid-catalyzed sol-gel reaction of tetraethyl orthosilicate (TEOS). FT-IR spectra and SEM images confirmed the presence of SiO_2_ in the SPAE matrix. With the increase in SiO_2_ amount, the IEC, and water uptake, the swelling ratio of the PEMs decreased, as expected. The proton conductivity reduced gradually from 93.4 to 76.9 mS cm^−1^ at room temperature as the loading amount of SiO_2_ was increased from 0 to 16 wt.%; however, the VO^2+^ permeability decreased dramatically after the incorporation of SiO_2_ and reached a minimum value of 2.57 × 10^−12^ m^2^ s^−1^ at 12 wt.% of SiO_2._ As a result, the H^+^/VO^2+^ selectivity achieved a maximum value of 51.82 S min cm^−3^ for the SPAE/SiO_2_-12. Moreover, the tensile strength and T_d-5%_ of SPAE/SiO_2_-12 were 37.03 MPa and 359.8 °C, respectively, which were much higher than those of pure SPAE (26.33 MPa and 344.8 °C). Therefore, the optimization of SiO_2_ amount is important for composite PEMs to balance the trade-off between proton conductivity and vanadium ion permeability, and SPAE/SiO_2_-12 is a promising candidate membrane for VRFB applications.

## Figures and Tables

**Table 1:** Basic properties of the SPAE and SPAE/SiO_2_ composite PEMs at room temperature.

Polymer	IEC (mmol g^−1^)	Water Uptake (%)	Swelling Ratio (%)	Tensile Strength (MPa)	Elongation at Break (%)
SPAE	1.97	25.6	13.3	26.33	24.8
SPAE/SiO_2_-4	1.90	23.5	11.6	27.28	21.5
SPAE/SiO_2_-8	1.82	21.6	10.5	31.01	18.3
SPAE/SiO_2_-12	1.76	18.3	9.6	37.03	15.0
SPAE/SiO_2_-16	1.70	15.2	8.7	29.13	9.7

**Table 2:** Properties of the SPAE and SPAE/SiO_2_ composite PEMs at room temperature.

Polymer	Thickness (μm)	σ (mS cm^−1^)	Ea (KJ mol^−1^)	Area Resistance (Ω cm^2^)	VO^2+^ Permeability (10^−12^ m^2^ s^−1^)	Ion Selectivity (10^3^ S min cm^−3^)
SPAE	58	93.4	16.31	0.71	7.62	20.42
SPAE/SiO_2_-4	53	88.2	15.58	0.87	6.11	24.06
SPAE/SiO_2_-8	55	83.9	15.44	0.93	4.31	32.44
SPAE/SiO_2_-12	51	79.9	15.00	1.17	2.57	51.82
SPAE/SiO_2_-16	50	76.9	14.03	1.59	3.64	35.21

## Data Availability

The data presented in this study are available on request from the corresponding author.

## Sample Availability

Samples of all compounds are available from the authors.
